# Head-Mounted Displays for Upper Limb Stroke Rehabilitation: A Scoping Review

**DOI:** 10.3390/jcm12237444

**Published:** 2023-11-30

**Authors:** Giulia Fregna, Chiara Paoluzzi, Andrea Baroni, Roberto Cano-de-la-Cuerda, Antonino Casile, Sofia Straudi

**Affiliations:** 1 Doctoral Program in Translational Neurosciences and Neurotechnologies, University of Ferrara, 44121 Ferrara, Italy; giulia.fregna@unife.it; 2 Department of Neuroscience and Rehabilitation, University of Ferrara, 44121 Ferrara, Italy; chiara.paoluzzi@edu.unife.it (C.P.); sofia.straudi@unife.it (S.S.); 3 Department of Neuroscience, Ferrara University Hospital, 44124 Ferrara, Italy; 4 Department of Physical Therapy, Occupational Therapy, Rehabilitation and Physical Medicine, Facultad de Ciencias de la Salud, Universidad Rey Juan Carlos, 28922 Alcorcon, Spain; roberto.cano@urjc.es; 5 Department of Biomedical and Dental Sciences and Morphofunctional Imaging, University of Messina, 98122 Messina, Italy

**Keywords:** head-mounted display, immersive virtual reality, upper extremity, stroke, motor recovery

## Abstract

Upper extremity (UE) paresis is one of the most frequent and disabling clinical consequences after stroke. Head-Mounted Displays (HMDs) are wearable virtual reality devices that seem effective in promoting the recovery of functional abilities by increasing adherence levels in this population. This scoping review is aimed at collecting available evidence on the use of HMD-based immersive virtual reality systems for UE rehabilitation treatment in stroke survivors. Four electronic bibliographic databases were consulted from inception until 18 January 2023. A total of 19 clinical trials in which HMDs were used as a clinical tool for increasing UE functioning, as a single intervention or in adjunct to other rehab treatments, were included; no restrictions were applied for UE paresis severity or stroke onset. The large majority of the clinical trials involved chronic stroke patients (15 out of 19), with a wide range of UE impairments. Overall, HMD use seemed to be well-tolerated and promising for increasing UE motor function in adult chronic stroke survivors, with benefits in subjects’ arm use and independence. The possibility of executing highly realistic and task-oriented movements appears to be promising in enhancing gesture relevance, thus promoting new motor strategies in a “virtual ecological way”. Across studies, we found a high heterogeneity in protocol design and a lack of reporting that prevents us drawing conclusions regarding potential subgroups of patients that could benefit more from HMD-based interventions or suggested treatment modalities.

## 1. Introduction

Stroke is the second leading cause of disability worldwide, affecting more than 12.2 million people every year [1]. According to the localization and extension of cerebral damage, patients experience sensorimotor and cognitive impairments that critically impact their quality of life [2,3]. Upper extremity (UE) paresis contralateral to the injured cerebral site is one of the most frequent and disabling clinical consequences after stroke, affecting more than 80% of acute patients and 40% of chronic ones [4]. Therefore, in relation to the crucial impact that UE function has in independently performing activities of daily living, UE sensorimotor recovery represents a key rehabilitation goal [5]. 

Neurorehabilitation treatment in post-stroke patients exploits neuroplasticity properties to maximize recovery and improve motor function [6,7]. Therapeutic interventions are based on principles that aim to enhance neuroplastic processes, including repetitive practice [8], a high training dosage [9], and task-oriented functional gestures [10].

Particularly, high-repetition dosage is key to improve UE function after stroke [11,12]. However, repetitive training can heavily impact patients’ adherence to treatment over time due to a lack of or decrease in motivation. Yet, it is well-known that patient engagement is associated with better rehabilitation outcomes [13]. Thus, a paramount goal of clinical research is to design novel rehabilitation interventions that promote high repetition dosage. Among the clinical means that can provide rehabilitation interventions in a challenging and engaging way, virtual reality tools represent a promising therapeutic option for boosting patients’ compliance over time. However, a terminology consensus on virtual reality affective constructs (such as motivation, engagement, and enjoyment) and related outcome measurements is still lacking [14].

Specifically, virtual reality (VR) is a simulation of a realistic or artificial environment created by a computer system, which allows the user to feel immersed and to interact with objects in that environment [15,16,17]. Mixed reality is instead a type of hybrid environment that blends the physical environment with virtual objects. It describes a linear continuum that ranges from real environments (reality) to fully virtual environments (virtuality). In mixed reality, the real and virtual contents allow for data contextualization; they provide real-time interactivity, and the content needs to be mapped and correlated with the 3D space. Within this continuum, we find augmented reality, which integrates virtual objects into real-life environments, usually using devices such as smartphones or wearable smart glasses. The real-life environment and the virtual objects interact through the augmented reality device in real time [18]. Thus, virtual, mixed, and augmented reality can be considered as parts of a single “Extended Reality” concept.

Among the VR tools, video games that require movements and physical effort to interact with the virtual environment are defined “exergames” [19] and, for the past decades, they have been gaining more and more attention as potential clinical tools [20,21]. Gaming consoles, such as Xbox^®^ and Nintendo Wii^®^, provide exercises in a stimulating context due to their capability of tracking and reproducing subjects’ movements on a screen in an interactive way [19]. Furthermore, given the devices’ ease of use and relatively low cost, exergames can be useful to perform rehabilitation treatments at patients’ homes in order to enhance and maintain the acquired motor abilities [22], also through telerehabilitation modalities [23]. Gaming platforms specifically developed for non-recreational purposes are defined as “serious games” [24].

The main concepts related to VR are immersion and interaction. Immersion refers to the extent to which the user perceives that she/he is in the virtual environment and is related to the design of the software and hardware, whereas interaction with the environment can be made through a variety of simple devices, such as a mouse or joystick, or more complex systems using cameras, sensors, or haptic (touch) feedback devices [25,26].

Depending on the “immersion” grade into the virtual environment experienced by the subject, VR devices can be divided into Non-Immersive VR (NIVR) tools and Immersive ones (IVR). NIVR tools include most gaming consoles where the virtual scenario is shown on a tv screen and is perceived by the subject concurrently with the real one. On the other hand, IVR devices allow the full immersion of the subject in the virtual environment [27] producing a feeling of “being there” (“presence” effect) [28]. 

Head-mounted displays (HMDs) and Cave automatic virtual environment (CAVE) systems are IVR tools. HMDs are wearable virtual reality broadcasting tools, significantly less expensive than CAVE systems that require a dedicated area where the interactive environment is placed [29]. Due the hardware’s cost-effectiveness, HMD-based immersive VR systems are presently the subject of intense research [30,31,32,33]. 

IVR has shown a positive effect on dexterity, gait performance, and dynamic balance in post-stroke subjects [34]; specifically, HMD-based VR rehabilitation systems seem effective at increasing functional abilities through higher motivation and adherence levels in this population type [35]. 

Recently, Hao et al. proved a greater clinical efficacy of IVR tools in UE treatment in post-stroke patients when compared to NIVR systems [36], probably due to the increased immersion provided that can amplify the user’s sensory experience, thus facilitating the interaction with the virtual world and increasing the relevance of the tasks, resulting in a greater motor transferability to the activities of daily life.

Concerning the use of HMDs as an IVR tool for UE functional recovery in post-stroke subjects, Marek et al. provided a relevant review of the literature available between 2019 and 2022 in this field [37]. However, comprehensive research based on a standardized, structured, recognized methodological conduction of what has been done so far is still lacking.

This is why this scoping review aims to collect available evidence on HMD use for UE rehabilitation treatment in stroke survivors is needed. In particular, the current review has the following goals:

- To collect all the available information on the clinical feasibility and effectiveness of HMD use for improving UE motor recovery in people after stroke in a bio-psycho-social view;
- To map relevant data related to the software and hardware characteristics of HMD interventions applied to UE treatment in stroke survivors so far;
- To track information on treatment protocols applied and therapeutic modalities proposed for HMD applications for UE rehabilitation in this population type;
- To identify stroke patients subgroups who could be more responsive to HMD use for increasing UE function.

## 2. Materials and Methods

### 2.1. Protocol and Registration

The protocol for the present scoping review was pre-registered in the Open Science Framework (OSF) registry https://osf.io/xsjzk/ (accessed on 16 May 2023). We used the PRISMA-ScR guidelines [38] as reference reporting (see Supplementary File S2).

A comprehensive search was performed to collect evidence about the efficacy of HMD-based IVR rehabilitation systems (as a single intervention or as an adjunct to other rehab treatments) for increasing UE functioning in post-stroke patients also when compared to other therapies or no interventions.

### 2.2. Eligibility Criteria

Clinical trials (Randomized Controlled Trials—RCTs, Non-Randomized Controlled Trials—nRCTs, and pre-post studies) were included, while scoping reviews and systematic reviews were excluded.

We included studies that followed the following inclusion criteria: (i) reference written in English; (ii) reference that presented original quantitative data; (iii) studies that included stroke patients with UE impairment; and (iv) studies describing the application of HMD-based IVR as a rehabilitation tool for UE. Exclusion criteria were (i) studies evaluating immersive virtual reality without using HMDs; (ii) clinical trials evaluating immersive virtual reality as a rehabilitation treatment, not for UE; and (iii) commentaries, editorials, or any published paper without primary data.

### 2.3. Types of Participants

The study population consisted of stroke patients with upper limb impairment; no restrictions were applied for stroke type (ischemic or hemorrhagic), time from injury, and cerebral area of damage. 

### 2.4. Types of Interventions and Outcome Measures

We included studies on post-stroke patients in which immersive virtual reality, presented by means of an HMDs, was used as a rehabilitation tool (alone or in combination with other interventions) for improving UE functional recovery, even compared to other rehabilitation techniques such as non-immersive/semi-immersive VR systems, conventional treatment or no interventions. We considered eligible multi-session studies that performed IVR treatments with various durations, intensities, and frequencies with time-dependent clinical follow-up. We applied no restriction on rehabilitation settings (i.e., hospitals or outpatient rehabilitation clinics). UE motor outcomes should have been measured through quantitative data from clinical tests (i.e., Fugl-Meyer Assessment, Action Research Arm Test) or derived by instrumental evaluations (i.e., fMRI, EEG, kinematic analysis).

### 2.5. Search Strategy

Articles published in peer-reviewed journals and pre-peer-review web publications were considered potentially eligible. Further, the bibliographies of the included articles were checked to find other potentially eligible studies. Author AB conducted literature searches of electronic bibliographic databases in PubMed, Web of Science, Science Direct, and Embase from inception to 18 January 2023.

The search strategy consisted of controlled vocabulary and primary keywords, such as “stroke”, “virtual reality”, “head-mounted display”, “upper extremity”. Refer to Supplementary File S3 for a detailed description of the search strategy.

### 2.6. Study Selection

**Figure 1:**
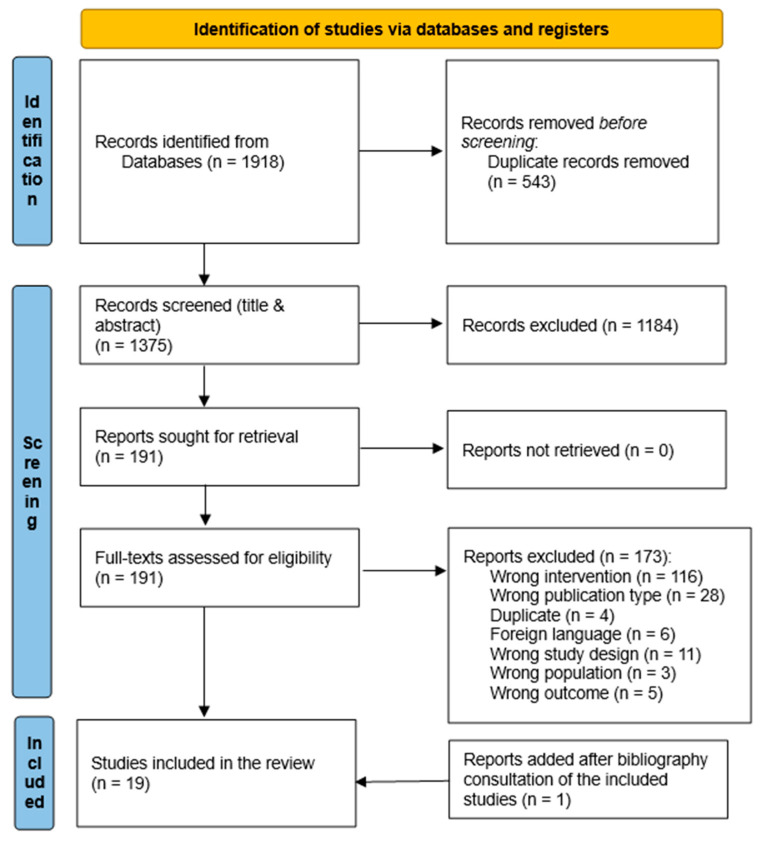
Flow chart of the identified studies according to the PRISMA-ScR Guidelines [38] and PRISMA 2020 Statement [60].

Titles and abstracts of shortlisted articles were screened for eligibility by two reviewers (GF and CP) independently and a third reviewer solved disagreements in study selection (AB and/or SS). Selected studies were then reviewed in full text by GF and CP and further selection discordances were addressed by AB or/and SS (Figure 1). Rayyan software was used for the selection process management (https://rayyan.ai/ accessed on 16 May 2023).

### 2.7. Data Extraction

Two authors independently uploaded data from included studies to a custom-designed data extraction form. The data chart included fields for author, publication year, study design, sample characteristics (stroke type, stroke timeframe, and age/sex/UE impairment severity of patients included), HMD description in terms of hardware and software features, intervention modalities applied (dosage, frequency, VR sessions’ time length, and co-interventions applied, if any), comparator details, outcomes measures, and main results found.

We performed a critical appraisal of included RCTs through the Cochrane Risk of Bias Tool (RoB) [39] while Joanna Briggs Institute (JBI) checklists were used to analyze the risk of bias for different study design projects (i.e., nRCT, case–control trials) [40]. Both tools are scientifically recognized means for assessing methodological quality of clinical studies.

Considering the high heterogeneity expected in terms of devices used, outcome measures, intervention modalities, and comparator(s) analyzed, a narrative description of the collected results was planned.

## 3. Results

The search strategy identified 1918 records; 191 passed beyond the title and abstract evaluation, of which 18 full texts met all inclusion criteria [41,42,43,44,45,46,47,48,49,50,51,52,53,54,55,56,57,58]. One article was added after bibliography consultation of the included studies [59]. We thus fully reviewed the text of 19 papers. The study identification process and the main exclusion reasons can be found in the PRISMA flowchart, reported in Figure 1.

Out of the 19 selected studies, 8 were RCTs and 11 were clinical studies that were sorted into NRCTs, Non-Controlled Clinical Trials (NCCTs), Case Series, and Case Reports. The included studies were published between 2008 and 2022 with the vast majority of them published after 2019 (16 out of 19). This pattern of results reflects the recent surge in interest in IVR as a rehabilitation tool. The majority of the studies were performed in Asia (8/19), followed by Europe (6/19) and the USA (5/19).

Across studies, we found a high heterogeneity in the type of HMD and treatment modalities (see Table 1 for the details of the included studies in terms of patients enrolled, intervention characteristics, and outcomes analyzed).

Almost all the clinical trials involved chronic stroke patients; only 4 studies tested an HMD-based IVR rehabilitation system in subacute stroke patients [45,49,53,59]. While not all studies reported the UE function prior to the treatment applied [47,49,51,54], HMD-based IVR systems were used even in subjects with severe UE paresis (FMA-UE score under 30) [45,53,56,58].

Among the studies that reported the specific type of HMD, almost all used either the Oculus Rift [42,44,46,47,54,55,56,57,58] or the HTC Vive [43,45,51,52,53,54,59]. Often, the hardware component comprised hand controllers [43,47,49,52,54,56,59] and/or other devices such as the Leap Motion Controller [42,44,45,46,53,54] or haptic tools [50,57]. From the software side, several authors used commercial VR platforms (i.e., Steam [43,51] and VIVE platforms [43], Tion; Human IT Solution [47], and Rehago software [49]) while some of them proposed virtual tasks through ad hoc-developed tools [41,42,44,45,48,50,52,53,54,55,56,57,58].

**Figure 2:**
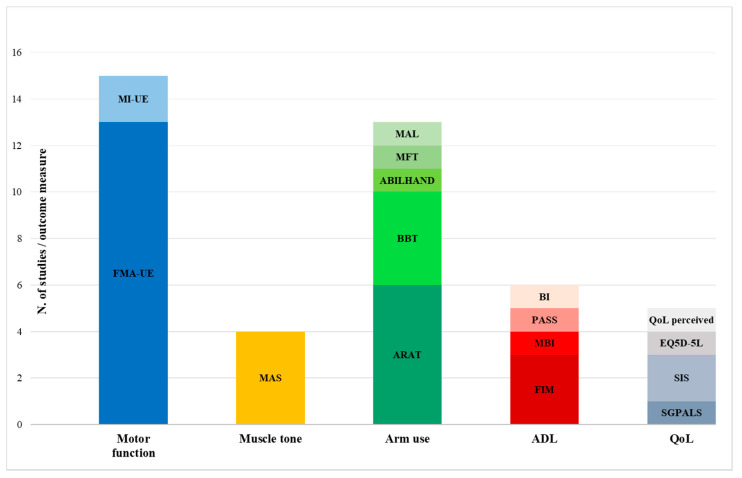
Number of studies that used the represented clinical tools for analyzing different ICF domains. All the studies which used the same clinical means are grouped in color clusters. Abbreviations: ADL = Activities Of Daily Living; QoL = Quality of Life; FMA-UE = Fugl-Meyer Assessment Upper Extremity; MI-UE = Motricity Index Upper Extremity; MAS = Modified Ashworth Scale; ARAT = Action Research Arm Test; BBT = Box And Block Test; MAL = Motor Activity Log; MFT = Manual Function Test; FIM = Functional Independence Measure; (M)BI = (Modified) Barthel Index; PASS = Performance Assessment of Self-care Skills; EQ5D-5L = EuroQol 5 Dimensions-5 Levels; SGPALS = Saltin–Grimby Physical Activity Level Scale; SIS = Stroke Impact Scale.

We found large differences between the included studies with respect to VR treatment modalities (session frequency, length, and dosage), with a minimum of 2.5 h of treatment proposed [57] to a maximum of 24 [58]; generally, a greater amount of sessions was associated with better clinical results [43,46,49]. Further, in consideration of the outcome measures analyzed, the HMD effect on different ICF domains has been investigated. Many authors have analyzed the role of HMD-based IVR systems on UE motor function (15/19) but also on arm use (9/19), less frequently on subjects’ independence in ADL (5/19) or quality of life (4/19) (Table 1 and Figure 2; discrepancies in study count between Table and Figure are related to authors who analyzed the same outcome through multiple clinical means).

### 3.1. Motor Function

All eight included RCTs found UE motor improvements in the patients enrolled [41,42,43,44,45,46,59]. Considering the studies that compared HMD use to conventional/occupational therapy, five out of seven RCTs found a statistically significantly greater increase in the FMA-UE score in patients treated with IVR training [42,43,44,45,46]. 

The positive effect of HMD use on UE paresis has also been reported by authors who have tested IVR in an NRCT [48], in case series [50,51,53,55,56], and in single-case reports [57,58]. The effect of HMDs on muscle tone has been rarely investigated so far, and limited clinical changes after treatment have been noticed by Hsu [42] and Vourvopoulus [57].

### 3.2. Arm Use

IVR motor rehabilitation through HMDs produced improvements in arm functioning greater than conventional treatment measured through different clinical tools (Table 2): through a statistically significant difference in the in ARAT score in the study of Ögün [46], and in BBT and MAL-QOM scores in the RCT of Hsu, when compared to usual mirror therapy [42]. Even for arm use, positive effects have also been noticed by authors that tested HMDs in series of patients [51,52].

### 3.3. ADL

Considering the role of HMDs in increasing patients’ abilities in independently performing activities of daily living (Table 2), some information is currently available from a few RCTs and some NRCTs. Encouraging results have been found by Ögün et al. in FIM, PASS-IADL, and PASS-BADL scores in the VR group compared to the control group through statistically significant improvements [46]. Positive effects have been recorded also by Chen [49] and Lee [52] in the patients assessed through the FIM and MBI scales, respectively.

### 3.4. Participation

No data on QoL are available from the RCTs included (Table 2); however, beneficial effects from HMD use have been reported in one Non-Controlled Clinical Trial (NCCT) [49], in Vourvopoulos’s Case series [55] and in a Case report [58].

### 3.5. Side-Effects and Sense of Presence

Cybersickness phenomena were reported occasionally in few of the selected studies. (Table 2). Some minor unpleasant events have been recorded and described as eye strain, nausea, and discomfort [43]. Vourvopoulos investigated also the presence and embodiment experienced by patients involved in IVR treatment, with Oculus Rift demonstrating a gradual greater immersion perceived during the sessions performed, particularly for body ownership feeling [58].

### 3.6. Risk of Bias

Considering the methodological quality of the studies involved (Table 3) and the related risk of bias, the high-quality reporting of devices used, dosage, and treatment modalities is crucial (and not always accurately described) [41,48,51,54], as well as a detailed characterization of the participants enrolled, in order to fully analyze the clinical outcomes recorded [47,49,51,54]. The relevant heterogeneity across studies in study design and methodological implications does not consent to a systematic comparison of the results obtained and a quantitative synthesis of them is still not possible.

## 4. Discussion

This is the first scoping review that has mapped the currently available literature on the use of HMD-based IVR systems for upper limb treatment in patients after stroke, providing a comprehensive overview of the devices applied, therapeutic modalities proposed, and results obtained in order to identify scientific needs for increasing high-quality treatments in this population type through a standardized, approved methodological conduction.

There is a growing interest in the use of IVR training on subjects with neurological disorders, as shown by the recent increase in published studies [34,35,61,62]. Indeed, the use of IVR appears to be a promising clinical tool for the rehabilitation of patients with neurological disability thanks to the high customizability of the virtual environment (e.g., in terms of tasks and contexts) and the greater cognitive stimulation linked to the immersion experienced by the subject. In particular, patients can train and learn new abilities in an enriched environment in a challenging and engaging modality, both of which are crucial elements for increasing rehabilitation outcomes [13]. The possibility of performing task-oriented gestures in a naturalistic manner during VR treatments may increase the motor transferability needed for inducing neuromotor improvements in patients after stroke [63,64] and could explain the encouraging results seen in the field [65]. On these bases, some authors have reviewed the literature on IVR training [34,35,36], and HMDs specifically [37], for UE recovery in stroke patients, showing valuable but partial information due to strict [37] or non-specific [34,35,36] search strategies, or derived by a non-structured, validated, methodological conduction [37].

As a result of our data collection, we can see that HMD-based IVR systems appear promising in improving UE neuromotor abilities after stroke, in accordance with what was already highlighted in the reviews conducted by Demeco, Patsaki, Hao, and Marek [34,35,36,37]. UE treatment by means of HMD has been investigated mostly in chronic stroke patients in the RCTs performed so far, and no consistent information is currently available on HMD effect in increasing UE function in the maximum recovery phase, thus in subacute stroke patients. Considering the large enrollment time needed in rehabilitation clinical trials involving subacute stroke subjects [66], it is plausible that in the next few years, further results on HMD clinical efficacy in this target population will be available. Almost only chronic stroke survivors were enrolled, and UE motor function was the main outcome in the majority of the studies involved (15/19). Concerning the possibility of significantly increasing UE motor abilities in the chronic phase (resulting in improvements in the FMA-UE or MI-scores), the gains found in the VR groups when compared to conventional treatments seem promising [42,43,44,45,46], although in some cases they were below the Minimal Clinical Important Difference cut-off. 

However, rehabilitation interventions in chronic stroke subjects are usually focused on function more than structural impairment, in relation to the windows of neurological recovery [67]. Accordingly, several studies have found improvements in the arm use of chronic patients treated with HMD-based IVR systems when compared to conventional treatments. In most cases, this difference was not significant, probably due to the small samples involved [41,47,59]. The potential usefulness of IVR training through HMDs in UE function is supported also by the positive results obtained in the ADL domain, with statistically significant improvements reported by Ögün in an RCT conducted in 65 stroke patients [46]. The possibility of executing highly realistic task-oriented movements could enhance the gesture relevance, thus promoting new motor strategies in a “virtual ecological way”. Indeed, the generalization of movements is a key element of motor learning processes [68], and the possibility of training complex gestures in IVR treatment could improve these mechanisms.

Lastly, HMD use seemed safe and well-tolerated by the patients with positive consequences on quality of life, as shown in the few studies included that analyzed this outcome [49,58]. As already reported in the literature, IVR application could increase patients’ motivation and quality of life [34], with beneficial effects also on depressive symptoms often reported in stroke survivors [69]. It is well known that “gamification” represents a key factor in rehab interventions, and challenging and stimulating tasks are essential for increasing patients’ compliance [70]. In this context, positive results have been found already in NIVR rehabilitation systems based, for example, on the Sony Playstation^®^ and Nintendo Wii^®^ consoles [70]. We thus expect that the greater “evasion effect” experienced by the patients in HMD-based IVR systems could further promote treatment adherence and sustain subjects’ engagement during the rehabilitation process. 

In the end, there are still not enough data to target specific patient subgroups that can benefit most from HMD use. Considering the primary studies included, positive effects on UE function have been reported in patients with different impairment severities: Mekbib enrolled patients with severe UE paresis (mean FMA-UE score 9.3 ± 3.8) [45], while Huang tested HMD application on mildly impaired subjects (mean FMA-UE score 49.4 ± 9.0) [43], with statistically significant UE motor improvements reported in both cases. Thus, HMDs for UE treatment in stroke survivors seem applicable, with no restriction related to UE paresis severity or time from stroke. However, there is still not enough information to fully understand the hardware characteristics needed for providing HMD treatment for patients who are not able to perform against-gravity movements. Some authors have used hand controllers to let patients interact with the virtual world while others have not, with clinical consequences in the definition of patients that could execute the movements requested. 

Concerning the treatment dosage proposed, a high heterogeneity was present across all included studies and a lack of reporting is notable. The overall mean duration of the administered HMD treatment was about 11 h but with a very high variance (2.5–24 h) [57,58], with the total of VR sessions ranging from 4 [51] to 42 [49], distributed across a variety of treatment lengths (2–9 weeks) [42,45,53]. Thus, no conclusions can be drawn on the suggested session frequency or minimal treatment length for achieving measurable clinical changes. 

Regarding the feasibility of HMD applications for UE treatment in stroke survivors, spare information on cybersickness symptoms has been provided so far but no serious side effects have been reported [43,55,56,58]. Further clinical tests are still needed to target software and hardware features that could maximize subjects’ immersion in VR context while avoiding uncomfortable perceptions. 

However, the provided information are partially influenced by the lack of reporting noticed in some of the papers included, in which an insufficient description of the sample enrolled [47,49,51,54] and/or the intervention applied [41,48,51,54] do not allow one to properly compare the observed results.

Finally, according to what observed in the clinical trials included, IVR training through HMDs seems helpful and feasible for increasing UE motor function in chronic stroke survivors, with benefits on patients’ arm use and no restrictions about UE impairment entity. 

Future studies are needed to define the best IVR setup needed for maximizing clinical usability and efficacy in stroke recovery. For instance, further clinical trials could highlight the role of haptic feedback in increasing subjects’ embodiment in the virtual environment and its effects on UE recovery. Moreover, the use of weight support tools that could provide information on HMD application in severely impaired patients, or even HMD as a means for home-based intervention in UE treatment, has been not extensively investigated. Considering the rapid growth of scientific publications in this field, the need for an early literature update is plausible.

This scoping review presents some limitations: particularly, the high heterogeneity across the included studies in terms of study design, comparator interventions, and treatment modalities applied do not allow a generalization of the findings, and precise clinical implications are not currently available. That said, this review represents a first systematic attempt to chart the existing literature on HMD use for UE recovery in post-stroke subjects, providing data on characteristics of the samples involved, devices used, and main clinical outcomes found. It describes a comprehensive overview of what has been done so far in order to clarify the clinical potential usefulness of HMD for UE treatment after stroke and highlight the scientific needs that have to be covered in the field.

## 5. Conclusions

In conclusion, the use of HMDs seems feasible and promising for increasing UE motor function in adult chronic stroke survivors, with benefits in subjects’ arm use and independence. Considering the high heterogeneity currently present across studies and the methodological quality assessed, no consistent information is available in terms of patients’ subgroups that could benefit more from IVR training or suggested HMD treatment protocols. Further, an improvement in the methodological conduction and reporting quality in the future clinical trials is needed in order to improve scientific and clinical recommendations in this field.

## Figures and Tables

**Table 1:** Characteristics of the included studies. For each study, information on patients enrolled, the HMD intervention applied, comparator analyzed (where present) and outcomes investigated are reported. Data on age and upper extremity impairment severity, measured through FMA-UE, MI, and ARAT score prior to treatment, are expressed as mean and standard deviation.

Study	Sample	Population	VR Dosage	Hardware& Software	Comparison	Outcome	Outcome Measures	Participants Analyzed
**RCTS**								
Crosbie, 2012 [41]	18 (M = 10, F = 8) VRG: 9 CG: 9	Chronic stroke patients VRG age 56.1 ± 14, MI-UE score 81.7 ± 9.4, ARAT score 51.3 ± 8.2 CG age 64.6 ± 7.4, MI-UE score 77.4 ± 19.5, ARAT score 47.3 ± 18.1	Three sessions/week for 3 weeks of 30–45 min each	HMD (model not mentioned), desktop computer, motion tracking system, sensors _________ -	CT	Motor function	MI-UE, ARAT	100%
Hsu, 2022 [42]	54 (M = 20, F = 32) VRG: 18 UMT: 18 COT: 18	Chronic stroke patients VRG age 52.9 ± 11.8, FMA-UE score 42.3 ± 14.3 UMT age 56.7 ± 11.5, FMA-UE score 32.1 ± 15.2; COT age 56.9 ± 13.0, FMA-UE score 34.5 ± 17.4	Two sessions/week for 9 weeks comprised of 30 min of VR mirror therapy + 20 min of CT	HMD Oculus Rift, personal computer-based desktop, Leap Motion Controller, two camera sensors _________ Unity software	UMT or COT	Motor function	FMA-UE, MAL, BBT, SWM, MAS	96%
Huang, 2020 [59]	18 (M = 15, F = 3). VRG: 9 CT: 9	Subacute/chronic stroke patients VRG age 59.5 ± 15.0, FMA-UE score 38.2 ± 19.6 CT age 55.3 ± 10.5, FMA-UE score 52.4 ± 10.1	Total of 20 sessions over 8 weeks of 30 min of VR + 60 min of CT + 60 min of OT each.	HMD HTC Vive, hand controllers, pc station _________ -	CT	Motor function, ADL	FMA-UE, BBT, FIM	100%
Huang, 2022 [43]	30 (M = 10, F = 20) VRG: 15 CG: 15	Chronic stroke patients VRG age 50.8± 12.3, FMA-UE score 49.4 ± 9.0; CG age 58.3 ± 11.2, FMA-UE score 44.5 ± 16.6	Total of 16 sessions of 60 min each, 2–3 days/week	HMD HTC Vive, hand controllers, two infrared laser emittent units _________ Steam or VIVE platform	COT	Motor function, biomarkers analysis, usability	FMA-UE, AROM, BDNF proteins, SSQ, Borg Scale of Perceived Exertion and self-reported measures on satisfaction and safety	100%
Lin, 2021 [44]	18 (M = 13; F = 5) VRG: 9 CG: 9	Chronic stroke patients VRG age 49.7 ± 13.4, FMA-UE score 43.4 ± 14.5 CG age 58.8 ± 9.6, FMA-UE score 28.3 ± 18.1	Two sessions/week for 9 weeks of 30 min of VR mirror therapy + 20 min of CT each	HMD Oculus Rift, Leap Motion controller _________ Unity software	UMT + CT	Motor function	FMA-UE	100%
Mekbib, 2021 [45]	23 (M = 17, F = 6) VRG: 12 CG: 11	Subacute stroke patients (<3 months) VRG age 52.2 ± 13.3, FMA-UE score 9.3 ± 3.8 CG age 61.0 ± 7.7, FMA-UE score 6.6 ± 2.2	Four sessions/week for 2 weeks of 60 min of VR training + 60 of OT each	HMD HTC Vive, Leap Motion controller, PC; HTC Vive tracking technology, HTC Vive link box _________ Unity software	OT	Motor function, ADL, Cerebral Activity	FMA-UE, BI, fMRI	100%
Ögün, 2019 [46]	65 (M = 51, F = 14) VRG: 33 CG: 32	Chronic stroke patients VRG age 61.5 ± 10.9, FMA-UE score 39.6 ± 8.8 CG age 59.8 ± 8.1, FMA-UE score 38.6 ± 8.8	Three sessions/week for 6 weeks of 60 min each	HMD Oculus Rift, Leap Motion controller, TV screen _________ -	CT + VR equipment without motor interaction	Motor function, ADL	FMA-UE, ARAT, FIM, PASS-IADL, PASS-BADL	100%
Song, 2021 [47]	10 (M = 6, F = 4) VRG: 5 CG: 5	Chronic stroke patients VRG age 64.2 ± 7.1 CG age 60.0 ± 10.9	Five sessions/week for 4 weeks of 30 min each of VR bilateral arm training + 60 min of CT at every session	HMD Oculus Rift, hand controllers, Notebook _________ Tion, Human IT Solution	Usual bilateral arm training + CT	Motor function, Proprioception, Muscle Activity, Cerebral Activity	MFT, two-point discrimination test, Proprioception test, Stereognosis test, EMG, EEG	100%
**NRCTs**								
Ma, 2008 [48]	8 (M = 4, F = 4) VRG: 2 CG: 2	Chronic stroke patients VRG age 59.5 ± 19.6, ARAT score 47.0 ± 11.2, MI score 84.0 ± 14.3 CG age 58.0 ± 16.4, ARAT score 55.5 ± 1.7, MI score 78.8 ± 3.5	Total of 10 sessions	HMD VR1280, desktop computer _________ -	CT	Motor function	MI-UE, ARAT	100%
**NCCTs**								
Chen, 2022 [49]	48 (M = 34, F = 14)	Subacute/chronic stroke patients	Total of 42 sessions, 30 min each	HMD Pico Neo 2, hand controllers _________ Rehago software	-	ADL, QoL	FIM, EQ5D-5L	100%
**Case Series**								
Connelly, 2009 [50]	7 *Sex data not reported*	Chronic stroke patients age 57 ± 18, stage 4 or 5 of the Hand Stage of Recovery of the Chedoke–McMaster Stroke Assessment, FMA-UE score 37 ± 8.8	Three sessions/week for 6 weeks of 30 min each	HMD Wide5, magnetic tracker, Pneuglove _________ Coin3D, CAVELib, Trackd-tool softwares	-	Motor function	FMA-UE	100%
Erhardsson, 2020 [51]	7 (M = 5, F = 2)	Chronic stroke patients age 60.6 ± 9.9	Total of 4–27 sessions in 10 weeks	HMD HTC Vive _________ Steam software	-	Motor function	ARAT, BBT and ABILHAND questionnaire, FMA-UE, MAS, SGPALS, Kinematics data	100%. Kinematics data from four patients
Lee, 2020 [52]	12 (M = 7, F = 5)	Chronic Stroke patients age 40.2 ± 17.8, ARAT score 23.9 ± 18.6	Total of 10 sessions, 2–3 sessions/week of 30 min each	HMD HTC Vive + hand controller _________ -	-	Motor function, usability	ARAT, BBT, MBI, self-reported usability questionnaire	100% (usability) 75% (Motor function)
Mekbib, 2020 [53]	8 (M = 6, F = 2)	Subacute stroke patients (<3 months) age 57.1 ± 4.5, FMA-UE score 7.5 ± 3.7	Total of 60 min of VR training + 60 min of CT per day (4 days/week) for 2 weeks	HMD HTC Vive, Leap Motion controller, PC; HTC Vive tracking technology, HTC Vive link box _________	-	Motor function, Cerebral Activity	FMA-UE, MRI	100%
Sramka, 2020 [54]	6 (4 for UE training) *Sex data not reported*	Not reported	Total of 11–12 sessions	HMD HTC Vive and Oculus Rift, hand controllers, Leap Motion controller _________ -	-	Motor function	Quantitative parameters (i.e., movement accuracy, limb orientation, movement speed)	100%
Vourvopoulos, 2019 [55]	4 (M = 3, F = 1)	Chronic stroke patients age 60.0 ± 5.8, FMA-UE score 31.8 ± 13.1	Eight sessions of 90 min each	HMD Oculus Rift _________ REINVENT system (VR-BCI intervention)	-	Motor function, quality of life, usability, Muscle Activity, Cerebral Activity	FMA-UE, MAS, SIS, SSQ, self-reported data on enjoyment and ease of use, EEG, EMG, MRI, TMS	100%
Weber, 2019 [56]	10 (M = 6, F = 4)	Chronic stroke patients age 54.1 ± 13.0, FMA-UE score 21.7 ± 8.2	Twelve sessions of 30 min each	HMD Oculus Rift, hand controllers, laptop computer, two tabletop infrared LED sensors _________ WiseMind	-	Motor function, usability	SSQ, SUS, FMA-UE, ARAT	100%
**Case Reports**								
Vourvopoulus, 2019 (2) [57]	1 male	A 60-year-old chronic stroke patient FMA-UE score 31	Ten sessions of 15 min each for 3 weeks	Oculus Rift + haptic feedback tools _________ NeuRow system (VR-BCI intervention)	-	Motor function, quality of life, motor-imagery capability, cognitive function, Cerebral Activity	FMA-UE, MAS, SIS, MoCA, VMIQ-2 questionnaire, EEG, fMRI	100%
Vourvopoulos, 2019 (3) [58]	1 male	A 69-year-old chronic stroke patient FMA-UE score 13	Sixteen sessions of 90 min each	HMD Oculus Rift; _________ REINVENT system (VR-BCI intervention)	-	Motor function, quality of life, Embodiment, Presence, Usability, Cerebral Activity	FMA-UE, SIS, SSQ, Presence Questionnaire, Embodiment Questionnaire, EEG data	100%

Abbreviations: M = males; F = females; VRG = Virtual Reality Group; CG = Control Group; CT = Conventional Therapy; OT = Occupational Therapy; COT = Conventional Occupational Therapy; UMT = Usual Mirror Therapy; ADL = Activities Of Daily Living; QoL = Quality of Life; FMA-UE = Fugl-Meyer Assessment Upper Extremity; MI-UE = Motricity Index Upper Extremity; AROM = Active Range Of Motion; MAS = Modified Ashworth Scale; ARAT = Action Research Arm Test; BBT = Box and Block Test; MAL = Motor Activity Log; MFT = Manual Function Test; FIM = Functional Independence Measure; (M)BI = (Modified) Barthel Index; PASS-BADL = Performance Assessment of Self-care Skills, Basic ADL; PASS-IADL = Performance Assessment of Self-care Skills, Instrumental ADL; SGPALS = Saltin–Grimby Physical Activity Level Scale; SIS = Stroke Impact Scale; EQ5D-5L = EuroQol 5 Dimensions-5 Levels; SSQ = Simulation Sickness Questionnaire; SUS = System Usability Scale; VMIQ2 = Vividness of Movement Imagery Questionnaire; BDNF = Brain-Derived Neurotrophic Factor; MoCA = Montreal Cognitive Assessment; SWM = Semmes–Weinstein monofilament; EMG = electromyography; EEG = electroencephalogram; TMS = Transcranial Magnetic Stimulation; (f)MRI = (functional) Magnetic Resonance Imaging.

**Table 2:** Results of HMD treatment. The effects of HMD intervention are presented per domains according to the International Classification of Functioning (ICF). Overall scores related to the pre-post intervention difference are described for each clinical scale as a mean value or numerical range in relation to the study design. Where possible, within- and between-group analysis are reported.

ICF Domain	Study	Intervention	Within Groups Results	Between Groups Results	Sample
**BODY FUNCTIONS**					
** Motor Function (FMA-UE, MI-UE)**					
RCTs					
	Crosbie, 2012 [41]	HMD VS CT	**MI-UE**: VRG from 81.7 to 84.9. CG from 77.4 to 85	No significant differences (*p* = 0.48)	18 VRG: 9 CG: 9
	Hsu, 2022 [42]	Oculus Rift + Leap Motion or UMT or COT	**FMA-UE**: VRG from 42.3 to 46.1 (*p <* 0.05) UMT from 32.1 to 34.4 (*p <* 0.05) COT from 34.5 to 35.1 (not significant)	Significant difference between VRG and COT (*p* = 0.03) in favor of VRG, not between VGR and UMT	52 VRG: 18 UMT: 17 COT: 17
	Huang, 2020 [59]	HTC Vive + hand controllers VS CT	**FMA-UE:** VRG from 38.22 to 46.78 (*p* = 0.01). CG from 52.44 to 55.56 (*p* = 0.02).	No significant differences (*p* = 0.08)	18 VRG: 9 CG: 9
	Huang, 2022 [43]	HTC Vive + hand controllers VS COT	**FMA-UE**: VRG from 49.40 to 52.47 (*p <* 0.05) COT from 44.47 to 45.53 (*p <* 0.05)	Significant difference in favor of VRG (*p ≤* 0.05)	30 VRG: 15 CG: 15
	Lin, 2021 [44]	Oculus Rift + Leap Motion VRMT VS UMT	**FMA-UE**: VRG from 43.4 to 46.7 (*p <* 0.05) CG from 28.3 to 29.2 (not significant)	Significant difference in favor of VRG (*p* = 0.03)	18 VRG: 9 CG: 9
	Mekbib, 2021 [45]	HTC Vive + Leap Motion VS OT	**FMA-UE**: VRG from 9.25 to 12.25 (*p <* 0.01) CG from 6.60 to 7.70 (not significant)	Significant difference in favor of VRG (*p* = 0.01)	23 VRG: 12 CG: 11
	Ögün, 2019 [46]	Oculus Rift + Leap Motion VS CT + only VR scenery	**FMA-UE**: VRG from 39.63 to 46.54 (*p <* 0.01) CG from 38.56 to 40.06 (*p <* 0.01)	Significant difference in favor of VRG (*p <* 0.01)	65 VRG: 33 CG: 32
NRCTs					
	Ma, 2008 [48]	VR 1280 + functional training VS functional training only	**MI-UE**: VRG improvement in all patients (*p* = 0.04) CG improvement in 2/4 patients (*p* = 0.14)		8 VRG: 4 CG: 4
Case Series					
	Connelly, 2009 [50]	Wide5 + Pneuglove	**FMA-UE**: From 37 to 43.1 (*p <* 0.01)		7
	Erhardsson, 2020 [51]	HTC Vive	**FMA-UE**: Improvements in 5/7 patients, gains between 3–5 points		7
	Mekbib, 2020 [53]	HTC Vive	**FMA-UE**: Improvements in 5/8 patients, gains between 1–11 points (*p* = 0.04)		8
	Vourvopoulus, 2019 [55]	Oculus Rift 1	**FMA-UE**: Improvements in 3/4 patients, gains between 1–6 points (not significant)		4
	Weber, 2019 [56]	Oculus Rift + hand controllers	**FMA-UE**: Improvements in 5/10 patients, gains between 1–5 points (not significant)		10
Case Reports					
	Vourvopoulos, 2019 (2) [57]	BCI through Oculus Rift + haptic feedback tools	**FMA-UE**: Gain of 9 points		1
	Vourvopoulos, 2019 (3) [58]	Oculus Rift	**FMA-UE**: Gain of 1 point		1
** Muscle tone (MAS)**					
RCTs					
	Hsu, 2022 [42]	Oculus Rift + Leap Motion VS UMT VS COT	**MAS:** No significant differences in all groups	Significant difference in wrist hypertonia between VRG and COT after treatment (*p* = 0.03) in favor of VRG	52 VRG: 18 UMT: 17 COT: 17
Case Series					
	Erhardsson, 2020 [51]	HTC Vive	**MAS:** No significant differences		7
	Vourvopoulus, 2019 [55]	Oculus Rift 1	**MAS:** No differences		4
Case Reports					
	Vourvopoulus, 2019 (2) [57]	BCI through Oculus Rift + haptic feedback tools	**MAS:** From 1+ to 2		1
**ACTIVITIES**					
** Arm use (ARAT, BBT, MAL, MFT, ABILHAND)**					
RCTs					
	Crosbie, 2012 [41]	HMD VS CT	**ARAT**: VRG from 51.3 to 52.8. CG from 47.3 to 50.2.	No significant differences (*p* = 0.14)	18 VRG: 9 CG: 9
	Hsu, 2022 [42]	Oculus Rift + Leap Motion VS UMT VS COT	**BBT**: Significant difference only in VRG, from 19.7 to 22.6 (*p <* 0.05)	Significant difference between VRG and UMT (*p* = 0.02) in favor of VRG	52 VRG: 18 UMT: 17 COT: 17
			**MAL-AOU**: Significant difference only in UMT, from 0.84 to 0.89 (*p <* 0.05)	No significant differences	
			**MAL-QOM**: Significant difference in UMT, from 0.91 to 0.95 (*p <* 0.05) and in VRG, from 1.19 to 1.31 (*p <* 0.05)	Significant difference between UMT and VRG (*p* = 0.05) in favor of VRG	
	Huang, 2020 [59]	HTC Vive + hand controllers VS CT	**BBT:** VRG from 17.44 to 29.67 (*p* = 0.12) CG from 29.67 to 35.44 (*p* = 0.10)	No significant differences (*p* = 0.42)	18 VRG; 9 CG: 9
	Ögün, 2019 [46]	Oculus Rift + Leap Motion VS CT + only VR scenery	**ARAT**: VRG from 32.81 to 41.15 (*p <* 0.01) CG from 30.84 to 32.09 (*p <* 0.01)	Significant difference in favor of VRG (*p <* 0.01)	65 VRG: 33 CG: 32
	Song, 2021 [47]	Oculus Rift + hand controllers VS conventional rehabilitation	**MFT**: Improvements in both VRG (*p* = 0.04) and CG (*p* = 0.04)	No significant differences	10 VRG: 5 CG: 5
NRCTs					
	Ma, 2008 [48]	VR 1280 + functional training VS functional training only	**ARAT**: Improvement in 1/4 patient (3 points) in VRG and in 2/4 patients of the CG (1 and 2 points)		8 VRG: 4 CG: 4
Case Series					
	Erhardsson, 2020 [51]	HTC Vive	**ARAT**: Improvements in 6/7 patients		7
			**BBT**: Improvements in 2/7 patients		
			**ABILHAND**: Improvements in 4/7 patients		
	Lee, 2020 [52]	HTC Vive + hand controllers	**ARAT**: From 22.3 to 31.1 (*p* = 0.03)		9
			**BBT**: From 11.2 to 19.6 (*p* = 0.01)		
	Weber, 2019 [56]	Oculus Rift + hand controllers	**ARAT**: Improvements in 2/10 patients, gains of 3 and 6 points (not significant)		10
** ADL (FIM, (M)BI, PASS-BADL, PASS-IADL)**					
RCTs					
	Huang, 2020 [59]		**FIM:** VRG from 112.67 to 108.56 (*p* = 0.25) CG from 99.33 to 104.11 (*p* = 0.12)	No significant differences (*p* = 0.06)	18 VRG: 9 CG: 9
	Mekbib, 2021 [45]	HTC Vive + Leap Motion + OT VS OT only	**BI**: VRG from 28.18 to 32.27 (*p* = 0.01) CG from 24.00 to 28.00 (*p* = 0.01)	No significant differences (*p* = 0.19)	23 VR: 12 CG: 11
	Ögün, 2019 [46]	Oculus Rift + Leap Motion VS CT + only VR scenery	**FIM**: VRG from 84.81 to 89.60 (*p <* 0.01) CG from 84.25 to 84.96 (*p <* 0.01)	Significant difference in favor of VRG (*p <* 0.01)	65 VRG: 33 CG: 32
			**PASS-BADL**: VRG from 1.46 to 1.84 (*p <* 0.01) CG from 1.53 to 1.56 (*p* = 0.51)	Significant difference in favor of VRG (*p <* 0.01)	
			**PASS-IADL**: VRG from 1.58 to 1.98 (*p <* 0.01) CG from 1.57 to 1.61 (*p* = 0.54)	Significant difference in favor of VRG (*p <* 0.01)	
NCCTs					
	Chen, 2022 [49]	Pico Neo 2 + Rehago	**FIM**: From 101.48 to 107.02, (*p <* 0.01)		48
Case Series					
	Lee, 2020 [52]	HTC Vive + hand controllers	**MBI**: From 90.4 to 93.0 (*p* = 0.04)		9
**PARTICIPATION**					
**QoL and Free time (EQ5D-5L, SGPALS, SIS)**					
NCCTs					
	Chen, 2022 [49]	Pico Neo 2	**EQ5D-5L**: From 12.52 to 11.62 (*p <* 0.03)		48
			**QoL perceived:** From 69.65 to 76.38 (*p <* 0.01)		
Case Series					
	Erhardsson, 2020 [51]	HTC Vive	**SGPALS**: No difference		7
	Vourvopoulos, 2019 [55]	Oculus Rift	**SIS**: Improvements in 1/4 patients, gain of 10 points (not significant)		4
Case Reports					
	Vourvopoulos, 2019 (3) [58]	Oculus Rift 1	**SIS**: Improvements of 30 points		1
** OTHERS: Feasibility (SSQ, SUS), Presence, Embodiment**					
RCTs					
	Huang, 2022 [43]	HTC Vive + hand controllers VS COT	**SSQ**: Mean score of 0.39. Total of 46.7% of patients experienced eye strain and 46.67% sweating, 26.6% of subjects experienced both symptoms		30 VRG: 15 CG: 15
Case Series					
	Vourvopoulos, 2019 [55]	Oculus Rift	**SSQ**: Changes after treatment: nausea subscale MD 0.13 (SD 1.46), oculomotor subscale MD -0.25 (SD 1.67) (not significant)		4
	Weber, 2019 [56]	Oculus Rift + hand controllers	**SUS**: Mean score of 76/100 (40–100)		10
			**SSQ**: From 1 to 1.6 after the first and the last session		
Case Reports					
	Vourvopoulos, 2019 (3) [58]	Oculus Rift 1	**SSQ**: No increases in nausea or oculo-motor sickness.		1
			**Presence:** Increasing trend across sessions		
			**Embodiment**: Increasing trend across sessions mostly for body ownership feeling		

Abbreviations: MD = Mean Difference; SD = Standard Deviation; VRG = Virtual Reality Group; CG = Control Group; CT = Conventional Therapy; OT = Occupational Therapy; COT = Conventional Occupational Therapy; UMT = Usual Mirror Therapy; ADL = Activities Of Daily Living; QoL = Quality of Life; FMA-UE = Fugl-Meyer Assessment Upper Extremity; MI-UE = Motricity Index Upper Extremity; MAS = Modified Ashworth Scale; ARAT = Action Research Arm Test; BBT = Box And Block Test; MAL-AOU = Motor Activity Log—Amount Of Use; MAL-QOM = Motor Activity Log—Quality Of Movement; MFT = Manual Function Test; FIM = Functional Independence Measure; (M)BI = (Modified) Barthel Index; PASS-BADL = Performance Assessment of Self-care Skills—Basic ADL; PASS-IADL = Performance Assessment of Self-care Skills—Instrumental ADL; EQ5D-5L = EuroQol 5 Dimensions-5 Levels; SGPALS = Saltin–Grimby Physical Activity Level Scale; SIS = Stroke Impact Scale; SSQ = Simulation Sickness Questionnaire; SUS = System Usability Scale.

**Table 3:** Critical appraisal of the included studies performed through the Cochrane and JBI tools accordingly to the study design. The numerical columns represent the different items of the checklists used for the Risk of Bias assessment (Cochrane RoB and JBI means).

**RCTs (Cochrane RoB)**											
**Study**	**1**	**2**		**3**	**4**		**5**	**6**		**7**	**Overall Score**
Crosbie, 2012 [41]	✓	✓		×	✓		✓	✓		✓	6/7
Hsu, 2022 [42]	✓	✓		×	✓		✓	✓		✓	6/7
Huang, 2020 [59]	?	?		×	?		✓	✓		✓	3/7
Huang, 2022 [43]	✓	✓		×	✓		✓	✓		✓	6/7
Lin, 2021 [44]	✓	✓		×	✓		✓	✓		✓	6/7
Mekbib, 2021 [45]	✓	✓		×	✓		×	✓		✓	5/7
Ögün, 2019 [46]	✓	?		✓	✓		×	✓		✓	5/7
Song, 2021 [47]	?	✓		×	?		?	✓		✓	3/7
**NRCTs (JBI checklist)**											
**Study**	**1**	**2**	**3**	**4**	**5**	**6**	**7**	**8**	**9**	**10**	**Overall Score**
Ma, 2008 [48]	✓	?	?	?	?	✓	×	✓	×	✓	4/10
**NCCT (JBI checklist)**											
**Study**	**1**	**2**	**3**		**4**	**5**	**6**	**7**	**8**	**9**	**Overall Score**
Chen, 2022 [49]	✓	NA	NA		×	✓	✓	NA	✓	✓	5/9
**Case Series (JBI checklist)**											
**Study**	**1**	**2**	**3**	**4**	**5**	**6**	**7**	**8**	**9**	**10**	**Overall Score**
Connelly, 2009 [50]	✓	✓	✓	?	?	×	✓	×	×	×	4/10
Erhardsson, 2020 [51]	✓	✓	✓	✓	✓	✓	✓	✓	?	✓	9/10
Lee, 2020 [52]	×	?	?	✓	?	✓	×	✓	?	✓	4/10
Mekbib, 2020 [53]	×	?	?	?	?	✓	×	✓	×	×	2/10
Sramka, 2020 [54]	×	×	×	?	?	×	×	×	×	×	0/10
Vourvopoulos, 2019 [55]	✓	?	?	?	?	×	×	✓	×	×	2/10
Weber, 2019 [56]	✓	✓	✓	?	×	✓	×	✓	✓	✓	7/10
**Case Reports (JBI checklist)**											
**Study**	**1**	**2**	**3**		**4**	**5**		**6**	**7**	**8**	**Overall Score**
Vourvopoulos, 2019 (2) [57]	✓	✓	✓		✓	✓		✓	?	✓	6/7
Vourvopoulos, 2019 (3) [58]	×	×	×		✓	✓		✓	✓	✓	5/7

Specifications: ✓ = low risk of bias, × = high risk of bias, ? = unclear risk, NA = Not Applicable.

## Supplementary Materials

The following supporting information can be downloaded at: https://www.mdpi.com/article/10.3390/jcm12237444/s1, Supplementary File S1: Abbreviations; Supplementary File S2: PRISMA-ScR; Supplementary File S3: Search String.

## Abbreviation

A full description of the acronyms used is available in Supplementary File S1.

## References

[1] Feigin V.L., Brainin M., Norrving B., Martins S., Sacco R.L., Hacke W., Fisher M., Pandian J., Lindsay P. (2022). World Stroke Organization (WSO): Global Stroke Fact Sheet 2022. Int. J. Stroke Off. J. Int. Stroke Soc..

[2] Chou C.-Y. (2015). Determinants of the Health-Related Quality of Life for Stroke Survivors. J. Stroke Cerebrovasc. Dis. Off. J. Natl. Stroke Assoc..

[3] Ramos-Lima M.J.M., Brasileiro I.d.C., de Lima T.L., Braga-Neto P. (2018). Quality of Life after Stroke: Impact of Clinical and Sociodemographic Factors. Clin. Sao Paulo Braz..

[4] Gresham G.E., Duncan P.W., Stason W.B., Adams H.P., Adelman A.M., Alexander D.N., Bishop D.S., Diller L., Donaldson N.E., Granger C.V. (1995). Post-Stroke Rehabilitation: Assessment, Referral and Patient Management. Am. Fam. Physician.

[5] Pollock A., Farmer S.E., Brady M.C., Langhorne P., Mead G.E., Mehrholz J., van Wijck F. (2014). Interventions for Improving Upper Limb Function after Stroke. Cochrane Database Syst. Rev..

[6] Sampaio-Baptista C., Sanders Z.-B., Johansen-Berg H. (2018). Structural Plasticity in Adulthood with Motor Learning and Stroke Rehabilitation. Annu. Rev. Neurosci..

[7] Rossini P.M., Calautti C., Pauri F., Baron J.-C. (2003). Post-Stroke Plastic Reorganisation in the Adult Brain. Lancet Neurol..

[8] French B., Thomas L.H., Coupe J., McMahon N.E., Connell L., Harrison J., Sutton C.J., Tishkovskaya S., Watkins C.L. (2016). Repetitive Task Training for Improving Functional Ability after Stroke. Cochrane Database Syst. Rev..

[9] Kwakkel G. (2009). Intensity of Practice after Stroke: More Is Better. Schweiz. Arch. Neurol. Psychiatr..

[10] Dobkin B.H. (2004). Strategies for Stroke Rehabilitation. Lancet Neurol..

[11] Lohse K.R., Lang C.E., Boyd L.A. (2014). Is More Better? Using Metadata to Explore Dose-Response Relationships in Stroke Rehabilitation. Stroke.

[12] Winstein C., Kim B., Kim S., Martinez C., Schweighofer N. (2019). Dosage Matters. Stroke.

[13] Langhorne P., Bernhardt J., Kwakkel G. (2011). Stroke Rehabilitation. Lancet Lond. Engl..

[14] Rohrbach N., Chicklis E., Levac D.E. (2019). What Is the Impact of User Affect on Motor Learning in Virtual Environments after Stroke? A Scoping Review. J. Neuroeng. Rehabil..

[15] Gutiérrez R.O., Galán Del Río F., Cano de la Cuerda R., Alguacil Diego I.M., González R.A., Page J.C.M. (2013). A Telerehabilitation Program by Virtual Reality-Video Games Improves Balance and Postural Control in Multiple Sclerosis Patients. NeuroRehabilitation.

[16] Luna-Oliva L., Ortiz-Gutiérrez R.M., Cano-de la Cuerda R., Piédrola R.M., Alguacil-Diego I.M., Sánchez-Camarero C., Martínez Culebras M.D.C. (2013). Kinect Xbox 360 as a Therapeutic Modality for Children with Cerebral Palsy in a School Environment: A Preliminary Study. NeuroRehabilitation.

[17] Ortiz-Gutiérrez R., Cano-de-la-Cuerda R., Galán-del-Río F., Alguacil-Diego I.M., Palacios-Ceña D., Miangolarra-Page J.C. (2013). A Telerehabilitation Program Improves Postural Control in Multiple Sclerosis Patients: A Spanish Preliminary Study. Int. J. Environ. Res. Public. Health.

[18] Campos E., Hidrogo I., Zavala G. (2022). Impact of Virtual Reality Use on the Teaching and Learning of Vectors. Front. Educ..

[19] Mat Rosly M., Mat Rosly H., Davis Oam G.M., Husain R., Hasnan N. (2017). Exergaming for Individuals with Neurological Disability: A Systematic Review. Disabil. Rehabil..

[20] Rüth M., Schmelzer M., Burtniak K., Kaspar K. (2023). Commercial Exergames for Rehabilitation of Physical Health and Quality of Life: A Systematic Review of Randomized Controlled Trials with Adults in Unsupervised Home Environments. Front. Psychol..

[21] Laver K.E., Lange B., George S., Deutsch J.E., Saposnik G., Crotty M. (2017). Virtual Reality for Stroke Rehabilitation. Cochrane Database Syst. Rev..

[22] Gelineau A., Perrochon A., Daviet J.-C., Mandigout S. (2022). Compliance with Upper Limb Home-Based Exergaming Interventions for Stroke Patients: A Narrative Review. J. Rehabil. Med..

[23] Laver K.E., Adey-Wakeling Z., Crotty M., Lannin N.A., George S., Sherrington C. (2020). Telerehabilitation Services for Stroke. Cochrane Database Syst. Rev..

[24] Sardi L., Idri A., Fernández-Alemán J.L. (2017). A Systematic Review of Gamification in E-Health. J. Biomed. Inform..

[25] Yavuzer G., Senel A., Atay M.B., Stam H.J. (2008). “Playstation Eyetoy Games” Improve Upper Extremity-Related Motor Functioning in Subacute Stroke: A Randomized Controlled Clinical Trial. Eur. J. Phys. Rehabil. Med..

[26] Cano-Mañas M.J., Collado-Vázquez S., Rodríguez Hernández J., Muñoz Villena A.J., Cano-de-la-Cuerda R. (2020). Effects of Video-Game Based Therapy on Balance, Postural Control, Functionality, and Quality of Life of Patients with Subacute Stroke: A Randomized Controlled Trial. J. Healthc. Eng..

[27] Slater M., Sanchez-Vives M.V. (2016). Enhancing Our Lives with Immersive Virtual Reality. Front. Robot. AI.

[28] Bohil C.J., Alicea B., Biocca F.A. (2011). Virtual Reality in Neuroscience Research and Therapy. Nat. Rev. Neurosci..

[29] Combe T., Chardonnet J.-R., Merienne F., Ovtcharova J. (2023). CAVE and HMD: Distance Perception Comparative Study. Virtual Real..

[30] Helou S., Khalil N., Daou M., El Helou E. (2023). Virtual Reality for Healthcare: A Scoping Review of Commercially Available Applications for Head-Mounted Displays. Digit. Health.

[31] Baashar Y., Alkawsi G., Wan Ahmad W.N., Alomari M.A., Alhussian H., Tiong S.K. (2023). Towards Wearable Augmented Reality in Healthcare: A Comparative Survey and Analysis of Head-Mounted Displays. Int. J. Environ. Res. Public. Health.

[32] Fregna G., Schincaglia N., Baroni A., Straudi S., Casile A. (2022). A Novel Immersive Virtual Reality Environment for the Motor Rehabilitation of Stroke Patients: A Feasibility Study. Front. Robot. AI.

[33] Casile A., Fregna G., Boarini V., Paoluzzi C., Manfredini F., Lamberti N., Baroni A., Straudi S. (2023). Quantitative Comparison of Hand Kinematics Measured with a Markerless Commercial Head-Mounted Display and a Marker-Based Motion Capture System in Stroke Survivors. Sensors.

[34] Demeco A., Zola L., Frizziero A., Martini C., Palumbo A., Foresti R., Buccino G., Costantino C. (2023). Immersive Virtual Reality in Post-Stroke Rehabilitation: A Systematic Review. Sensors.

[35] Patsaki I., Dimitriadi N., Despoti A., Tzoumi D., Leventakis N., Roussou G., Papathanasiou A., Nanas S., Karatzanos E. (2022). The Effectiveness of Immersive Virtual Reality in Physical Recovery of Stroke Patients: A Systematic Review. Front. Syst. Neurosci..

[36] Hao J., He Z., Yu X., Remis A. (2023). Comparison of Immersive and Non-Immersive Virtual Reality for Upper Extremity Functional Recovery in Patients with Stroke: A Systematic Review and Network Meta-Analysis. Neurol. Sci. Off. J. Ital. Neurol. Soc. Ital. Soc. Clin. Neurophysiol..

[37] Marek K., Zubrycki I., Miller E. (2022). Immersion Therapy with Head-Mounted Display for Rehabilitation of the Upper Limb after Stroke-Review. Sensors.

[38] Tricco A.C., Lillie E., Zarin W., O’Brien K.K., Colquhoun H., Levac D., Moher D., Peters M.D.J., Horsley T., Weeks L. (2018). PRISMA Extension for Scoping Reviews (PRISMA-ScR): Checklist and Explanation. Ann. Intern. Med..

[39] Higgins J.P.T., Altman D.G., Gøtzsche P.C., Jüni P., Moher D., Oxman A.D., Savovic J., Schulz K.F., Weeks L., Sterne J.A.C. (2011). The Cochrane Collaboration’s Tool for Assessing Risk of Bias in Randomised Trials. BMJ.

[40] JBI Critical Appraisal Tools|JBI. https://jbi.global/critical-appraisal-tools.

[41] Crosbie J.H., Lennon S., McGoldrick M.C., McNeill M.D.J., McDonough S.M. (2012). Virtual Reality in the Rehabilitation of the Arm after Hemiplegic Stroke: A Randomized Controlled Pilot Study. Clin. Rehabil..

[42] Hsu H.-Y., Kuo L.-C., Lin Y.-C., Su F.-C., Yang T.-H., Lin C.-W. (2022). Effects of a Virtual Reality-Based Mirror Therapy Program on Improving Sensorimotor Function of Hands in Chronic Stroke Patients: A Randomized Controlled Trial. Neurorehabil. Neural Repair.

[43] Huang C.-Y., Chiang W.-C., Yeh Y.-C., Fan S.-C., Yang W.-H., Kuo H.-C., Li P.-C. (2022). Effects of Virtual Reality-Based Motor Control Training on Inflammation, Oxidative Stress, Neuroplasticity and Upper Limb Motor Function in Patients with Chronic Stroke: A Randomized Controlled Trial. BMC Neurol..

[44] Lin C.W., Kuo L.C., Lin Y.C., Su F.C., Lin Y.A., Hsu H.Y. (2021). Development and Testing of a Virtual Reality Mirror Therapy System for the Sensorimotor Performance of Upper Extremity: A Pilot Randomized Controlled Trial. IEEE Access.

[45] Mekbib D.B., Debeli D.K., Zhang L., Fang S., Shao Y., Yang W., Han J., Jiang H., Zhu J., Zhao Z. (2021). A Novel Fully Immersive Virtual Reality Environment for Upper Extremity Rehabilitation in Patients with Stroke. Ann. N. Y. Acad. Sci..

[46] Ögün M.N., Kurul R., Yaşar M.F., Turkoglu S.A., Avci Ş., Yildiz N. (2019). Effect of Leap Motion-Based 3D Immersive Virtual Reality Usage on Upper Extremity Function in Ischemic Stroke Patients. Arq. Neuropsiquiatr..

[47] Song Y.-H., Lee H.-M. (2021). Effect of Immersive Virtual Reality-Based Bilateral Arm Training in Patients with Chronic Stroke. Brain Sci..

[48] Ma M., Bechkoum K. Serious Games for Movement Therapy after Stroke. Proceedings of the 2008 IEEE International Conference on Systems, Man and Cybernetics.

[49] Chen C.-H., Kreidler T., Ochsenfahrt A. (2022). Rehago—A Home-Based Training App Using Virtual Reality to Improve Functional Performance of Stroke Patients with Mirror Therapy and Gamification Concept: A Pilot Study. Stud. Health Technol. Inform..

[50] Connelly L., Stoykov M.E., Jia Y., Toro M.L., Kenyon R.V., Kamper D.G. Use of a Pneumatic Glove for Hand Rehabilitation Following Stroke. Proceedings of the 2009 Annual International Conference of the IEEE Engineering in Medicine and Biology Society.

[51] Erhardsson M., Alt Murphy M., Sunnerhagen K.S. (2020). Commercial Head-Mounted Display Virtual Reality for Upper Extremity Rehabilitation in Chronic Stroke: A Single-Case Design Study. J. Neuroeng. Rehabil..

[52] Lee S.H., Jung H.-Y., Yun S.J., Oh B.-M., Seo H.G. (2020). Upper Extremity Rehabilitation Using Fully Immersive Virtual Reality Games With a Head Mount Display: A Feasibility Study. PM R.

[53] Mekbib D.B., Zhao Z., Wang J., Xu B., Zhang L., Cheng R., Fang S., Shao Y., Yang W., Han J. (2020). Proactive Motor Functional Recovery Following Immersive Virtual Reality-Based Limb Mirroring Therapy in Patients with Subacute Stroke. Neurother. J. Am. Soc. Exp. Neurother..

[54] Sramka M., Lacko J., Ruzicky E., Masan J. (2020). Combined Methods of Rehabilitation of Patients after Stroke: Virtual Reality and Traditional Approach. Neuro Endocrinol. Lett..

[55] Vourvopoulos A., Pardo O.M., Lefebvre S., Neureither M., Saldana D., Jahng E., Liew S.-L. (2019). Effects of a Brain-Computer Interface With Virtual Reality (VR) Neurofeedback: A Pilot Study in Chronic Stroke Patients. Front. Hum. Neurosci..

[56] Weber L.M., Nilsen D.M., Gillen G., Yoon J., Stein J. (2019). Immersive Virtual Reality Mirror Therapy for Upper Limb Recovery After Stroke: A Pilot Study. Am. J. Phys. Med. Rehabil..

[57] Vourvopoulos A., Jorge C., Abreu R., Figueiredo P., Fernandes J.-C., Bermúdez i Badia S. (2019). Efficacy and Brain Imaging Correlates of an Immersive Motor Imagery BCI-Driven VR System for Upper Limb Motor Rehabilitation: A Clinical Case Report. Front. Hum. Neurosci..

[58] Vourvopoulos A., Marin-Pardo O., Neureither M., Saldana D., Jahng E., Liew S.-L., Chen J.Y.C., Fragomeni G. (2019). Multimodal Head-Mounted Virtual-Reality Brain-Computer Interface for Stroke Rehabilitation. Virtual, Augmented and Mixed Reality. Multimodal Interaction, Proceedings of the 11th International Conference, VAMR 2019, Held as Part of the 21st HCI International Conference, HCII 2019.

[59] Huang L.-L., Chen M.-H. (2020). Effectiveness of the Immersive Virtual Reality in Upper Extremity Rehabilitation. Cross-Cultural Design. Applications in Health, Learning, Communication, and Creativity, Proceedings of the 12th International Conference, CCD 2020, Held as Part of the 22nd HCI International Conference, HCII 2020.

[60] Page M.J., McKenzie J.E., Bossuyt P.M., Boutron I., Hoffmann T.C., Mulrow C.D., Shamseer L., Tetzlaff J.M., Akl E.A., Brennan S.E. (2021). The PRISMA 2020 Statement: An Updated Guideline for Reporting Systematic Reviews. BMJ.

[61] Pau M., Cocco E., Arippa F., Casu G., Porta M., Menascu S., Achiron A., Kalron A. (2023). An Immersive Virtual Kitchen Training System for People with Multiple Sclerosis: A Development and Validation Study. J. Clin. Med..

[62] Yun S.J., Hyun S.E., Oh B.-M., Seo H.G. (2023). Fully Immersive Virtual Reality Exergames with Dual-Task Components for Patients with Parkinson’s Disease: A Feasibility Study. J. Neuroeng. Rehabil..

[63] Kim A., Schweighofer N., Finley J.M. (2019). Locomotor Skill Acquisition in Virtual Reality Shows Sustained Transfer to the Real World. J. Neuroeng. Rehabil..

[64] Levac D.E., Huber M.E., Sternad D. (2019). Learning and Transfer of Complex Motor Skills in Virtual Reality: A Perspective Review. J. Neuroeng. Rehabil..

[65] Chen J., Or C.K., Chen T. (2022). Effectiveness of Using Virtual Reality-Supported Exercise Therapy for Upper Extremity Motor Rehabilitation in Patients With Stroke: Systematic Review and Meta-Analysis of Randomized Controlled Trials. J. Med. Internet Res..

[66] Geed S., Feit P., Edwards D.F., Dromerick A.W. (2021). Why Are Stroke Rehabilitation Trial Recruitment Rates in Single Digits?. Front. Neurol..

[67] Saes M., Mohamed Refai M.I., van Beijnum B.J.F., Bussmann J.B.J., Jansma E.P., Veltink P.H., Buurke J.H., van Wegen E.E.H., Meskers C.G.M., Krakauer J.W. (2022). Quantifying Quality of Reaching Movements Longitudinally Post-Stroke: A Systematic Review. Neurorehabil. Neural Repair.

[68] Krakauer J.W., Mazzoni P., Ghazizadeh A., Ravindran R., Shadmehr R. (2006). Generalization of Motor Learning Depends on the History of Prior Action. PLoS Biol..

[69] Kiper P., Przysiężna E., Cieślik B., Broniec-Siekaniec K., Kucińska A., Szczygieł J., Turek K., Gajda R., Szczepańska-Gieracha J. (2022). Effects of Immersive Virtual Therapy as a Method Supporting Recovery of Depressive Symptoms in Post-Stroke Rehabilitation: Randomized Controlled Trial. Clin. Interv. Aging.

[70] Tosto-Mancuso J., Tabacof L., Herrera J.E., Breyman E., Dewil S., Cortes M., Correa-esnard L., Kellner C.P., Dangayach N., Putrino D. (2022). Gamified Neurorehabilitation Strategies for Post-Stroke Motor Recovery: Challenges and Advantages. Curr. Neurol. Neurosci. Rep..

