# Supplementary material for: Head-Mounted Displays for Upper Limb Stroke Rehabilitation: A Scoping Review
Source: J Clin Med. 2023 Nov 30;12(23):7444. doi: 10.3390/jcm12237444 (PMC10706861; doi:10.3390/jcm12237444)
Supplement: Supplementary file 1 [file jcm-12-07444-s001.zip › Supplement File S1.pdf]

Head-mounted displays for upper limb stroke rehabilitation: a scoping review.

Abbreviation	Meaning
ADL	Activities of Daily Living
ARAT	Action Research Arm Test
AROM	Active Range Of Motion
BBT	Box and Block Test
BDNF	Brain-Derived Neurotrophic Factor
BI	Barthel Index
CAVE	Cave Automatic Virtual Environment
CG	Control Group
COT	Conventional Occupational Therapy
CT	Conventional Therapy
EEG	Electroencephalography
EMG	Electromyography
EQ5D-L	EuroQol 5 Dimensions-5 Levels
F	Females
FIM	Functional Independence Measure
FMA-UE	Fugl-Meyer Assessment Upper Extremity
(f)MRI	(functional) Magnetic Resonance Imaging
HMD	Head Mounted Display
ICF	International Classification of Functioning
IVR	Immersive Virtual Reality
M	Males
MAL	Motor Activity Log
MAL-AOU	Motor Activity Log - Amount Of Use
MAL-QOM	Motor Activity Log - Quality Of Movement
MAS	Modified Ashworth Scale
(M)BI	(Modified) Barthel Index
MD	Mean Difference
MFT	Manual Function Test
MI	Motricity Index
MoCA	Montreal Cognitive Assessment
NA	Not Applicable

NCCT	Non-Controlled Clinical Trial
NIVR	Non-Immersive Virtual Reality
NRCT	Non-Randomized Clinical Trial
OT	Occupational Therapy
PASS-BADL	Performance Assessment of Self-care Skills, Basic ADL
PASS-IADL	Performance Assessment of Self-care Skills, Instrumental ADL
QoL	Quality of Life
RCT	Randomized Controlled Trial
RoB	Risk of Bias
SD	Standard Deviation
SIS	Stroke Impact Scale
SGPALS	Saltin-Grimby Physical Activity Level Scale
SUS	System Usability Scale
SWM	Semmes-Weinstein monofilament
TMS	Transcranial Magnetic Stimulation
UE	Upper Extremity
UMT	Usual Mirror Therapy
VR	Virtual Reality
VRG	Virtual Reality Group

Table S1 – list of the acronyms used.
