# Supplementary material for: Head-Mounted Displays for Upper Limb Stroke Rehabilitation: A Scoping Review
Source: J Clin Med. 2023 Nov 30;12(23):7444. doi: 10.3390/jcm12237444 (PMC10706861; doi:10.3390/jcm12237444)
Supplement: Supplementary file 1 [file jcm-12-07444-s001.zip › Supplement File S3.pdf]

**Supplement C: Search String**

# Head-mounted displays for upper limb stroke rehabilitation: a scoping review.

**PUBMED**

(((((((stroke[MeSH Terms]) OR (stroke[Title/Abstract])) OR (Cerebrovascular Accident[Title/Abstract])) OR (CVA[Title/Abstract])) OR (Hemiparesis[Title/Abstract])) OR (Hemiplegia[Title/Abstract])) AND (((((virtual reality[MeSH Terms]) OR (virtual reality[Title/Abstract])) OR (immersive virtual reality[Title/Abstract])) OR (Smart Glasses[MeSH Terms])) OR (Head Mounted Display[Title/Abstract]))) AND ((((Upper Extremity[MeSH Terms]) OR (upper extremit*[Title/Abstract])) OR (upper limb[Title/Abstract])) OR (arm[Title/Abstract]))

**EMBASE**

'cerebrovascular disease':ab,kw,ti OR 'cerebrovascular accident':ab,kw,ti OR 'stroke patient':ab,kw,ti OR 'hemiparesis':ab,kw,ti OR 'hemiplegia':ab,kw,ti

AND

'virtual reality':ab,kw,ti OR 'head-mounted display':ab,kw,ti OR 'smart glasses':ab,kw,ti OR 'immersive virtual reality':ab,kw,ti OR 'virtual reality head mounted display':ab,kw,ti

AND

'upper limb':ab,kw,ti

**WEB of SCIENCE**

(((((TS=(stroke)) OR TS=(Cerebrovascular Accident)) OR TS=(CVA)) OR TS=(Hemiparesis)) OR TS=(Hemiplegia)) OR TS=(Cerebrovascular Disease)

AND

(((TS=(Virtual reality)) OR TS=(immersive virtual reality)) OR TS=(Smart Glasses)) OR TS=(Head Mounted Display)

AND

((TS=(upper extremit*)) OR TS=(upper limb)) OR TS=(arm)

**SCIENCEDIRECT**

(stroke OR cerebrovascular accident OR hemiparesis OR hemiplegia) AND (virtual reality OR head mounted display OR smart glasses) AND (upper limb OR upper extremity)
